# A case report of total skin photon radiation therapy for cutaneous epitheliotropic lymphoma in a dog

**DOI:** 10.1186/s12917-019-2105-4

**Published:** 2019-11-09

**Authors:** Michael A. Deveau, Megan Sutton, Courtney Baetge, Alison B. Diesel

**Affiliations:** 1 Department of Small Animal Clinical Sciences, College of Veterinary Medicine & Biomedical Sciences, Texas A&M University, 4474 TAMU, College Station, TX 77843-4474 USA; 2 Mission Veterinary Specialty and Emergency, San Antonio, TX 78249 USA

**Keywords:** Dogs, Cutaneous lymphoma, Radiation, Total skin

## Abstract

**Background:**

Total skin electron beam radiation therapy (TSEBT) is an effective treatment for primary diffuse cutaneous lymphomas in humans. While several techniques exist, they all require significant commitment of staff time and resources. In veterinary medicine, canine-specific techniques and strategies have been adapted and delivered but deemed not “realistically” clinically implementable given the time commitment of over 2.5 h plus per fraction or have been relegated to palliative intent. Leveraging these technologies of helical tomotherapy and 3D printing, we developed and clinically implemented a radiotherapeutic treatment strategy for the management of medically refractory diffuse cutaneous lymphoma in the dog.

**Case presentation:**

A 13.5-year-old female spayed Bichon Frise presented to the Oncology service at Texas A&M University, College of Veterinary Medicine due to the progression of diffuse cutaneous epitheliotropic lymphoma (CEL) that had failed medical management. Twenty-seven gray were delivered to the patient with a treatment time requirement under 40 min including real time monitoring of anesthesia during setup and treatment. A partial response was noticeable after four fractions and the tumor completely regressed progressively over the entire treated area by the end of therapy. A grade 1 lethargy, fatigue, weight loss, and oral mucositis and grade 2 alopecia, nail/claw changes, pruritus, scaling, anorexia, and diarrhea were noted during treatment. Additionally, a grade 3 thrombocytopenia developed after fraction eight requiring a treatment interruption of 6 weeks and prescription modification prior to treatment continuation and completion. From the beginning of total skin photon radiation therapy (TSPT) treatment until the time of the patient was euthanized unrelated to cutaneous epitheliotropic lymphoma (123 days), only one new lesion on the head was identified and confirmed by histopathology within the treated fields.

**Conclusions:**

The proposed technique is an acceptable alternative to TSEBT that is actually clinically implementable within a palliative or definitive setting and clinical constraints, however further testing and refinement is needed to reduce hematological complications and to confirm and expand on preliminary findings.

## Background

Cutaneous epitheliotropic lymphoma (CEL) in the dog is an uncommon neoplastic condition with a poor prognosis. Many treatment strategies have been reported, but in general, the prognosis is poor especially in those patients with advanced or diffuse cutaneous presentations [1–6]. In humans, total skin electron beam radiation therapy (TSEBT) is considered the primary treatment modality for late stage (T3) as well as early stage (T1–2) therapy refractory mycosis fungoides [7, 8]. While various permutations of techniques exist, one of the most common being the Stanford 6-dual field technique, several challenges exist requiring expertise and an established infrastructure to successfully and safely implement [9, 10].

Briefly, in order to deliver the treatment dose in the Stanford 6-dual field technique, the position of the patient is changed six times which combines two overlapping treatment fields per position for a total of 12 fields during one treatment fraction. Treatment of the primary fields is split over 2 days (three dual fields per day) with four treatment days per week for 6 to 9 weeks. Uniformity of dose across and along the patient’s longitudinal axis is of paramount importance, however, the use of overlapping electron fields causes dose variations upwards of +/− 15% secondary to patient motion and self-shielding [11]. Additionally, extra time is also required to “patch” treat areas missed by the primary fields such as the perineum, scalp, and sanctuary areas under the breasts or superfluous skin folds [12].

In veterinary medicine, only a limited number of patient cases utilizing radiation therapy for treating diffuse cutaneous lymphoma are reported [13–15]. A similar TSEBT 6-dual field technique was developed and delivered to a cadaver dog utilizing the assistance of a medical physicist with expertise in TSEBT [16]. Although determined mechanistically feasible, the time commitment for the patient and the technical staff was not realistically clinically implementable. It was reported that several hours were required to deliver prescription dose to each of the 12 treatment sub fields to a cadaver and this was ignoring real-time patient treatment factors such as anesthetic induction and monitoring or the impact on dose distribution due to motion and setup uncertainty.

Helical tomotherapy is a form of intensity-modulated radiation therapy (IMRT), in which all the functional parts of a 6-MV linear accelerator are installed on a ring-style gantry and rotated around the patient as the patient translates through the bore [17]. In veterinary medicine, helical tomotherapy offers several potential advantages for total skin treatments over other conventional C-arm style linacs. Firstly, the patients are anesthetized and immobilized for treatment in a natural anatomic position. Secondly, helical tomotherapy is equipped with an onboard single-row CT detector allowing for megavoltage 3D volumetric imaging and post treatment dose reconstruction. Thirdly, helical tomotherapy can irradiate extended volumes with a treatment length of up to 160 cm in a single setup position. Finally, the plan is optimized utilizing a modern treatment planning workflow, offering the potential to reduce dose heterogeneity and the need for “patch fields”. With helical tomotherapy a highly conformal and homogeneous dose distribution can be achieved but the combination of air to skin transition, obliquity in treatment beamlets, and the motion impact of breathing make surface doses difficult to accurately calculate and predict. A few studies have previously reported on the theoretical and clinical use of total skin photon radiation therapy (TSPT) in humans, but the technique as described (the use of a wet suit as a physical bolus) doesn’t translate well across species [18–20].

Three dimensional printing is revolutionizing the manufacturing industry by offering a potential solution via the ability to rapidly create medical imaging and computer-aided design models. While several variations of 3D printing techniques exist, extrusion-based 3D printing has been used by researchers to engineer eloquent, individualized solutions to common clinical/patient challenges. In veterinary radiation oncology, the technique offers the ability to create spatially unique or patient-specific structures by utilizing deposition tools that can extrude a variety of near-tissue equivalent materials [21].

Leveraging these technologies, we developed a treatment strategy for managing diffuse manifestations of CEL (or any diffuse cutaneous condition responsive to radiation therapy). Here, we report the successful and first clinical implementation of TSPT treated with helical tomotherapy for a case of therapy-refractory CEL in veterinary medicine.

## Case presentation

In June, a 13.5-year-old female spayed Bichon Frise presented to the Oncology service at Texas A&M University, College of Veterinary Medicine & Biomedical Sciences due to the progression of diffuse CEL. Approximately 8 months before presentation, the patient was seen at Pennsylvania State University, College of Veterinary Medicine and conservatively managed with antibiotic therapy for a mildly erythematous and pruritic, noduloplaque skin rash over her right caudal thorax. Concurrently while on therapy, several new multi-focal ulcerative lesions presented. Skin scrapes and a punch biopsy were performed and findings confirmed (dermatopathologist’s histopathological description consistent with, no special stains were submitted) epitheliotropic lymphoma. In April, the patient was started on L-asparaginase/CCNU/Prednisone/Denamarin protocol with subjective clinical improvement. At the client’s request, the patient was referred to Hope Veterinary Specialist to concurrently participate in a clinical trial utilizing monoclonal T cell therapy in combination with traditional chemotherapy. In May, CCNU was delayed secondary to hepatotoxicity. While on CCNU break, several new ulcerative lesions were noted on the right thorax, right ventral tail base, right perianal and vulvar region, and ventral thorax. The patient was started on Cyclophosphamide/Hydroxydaunorubicin/Vincristine/Prednisone (CHOP) receiving only one administration of vincristine prior to being switched to a modified Mechlorethamine/Vinblastine/Procarbazine/Prednisone (MOPP-based) protocol after continued disease progression. After the patient received two cycles of the modified MOPP-based protocol with no apparent response, the client was referred to Texas A&M University for participation in the leukotoxin (Leukothera®) clinical trial. On presentation the physical exam was unremarkable with the exception of the skin which revealed a sparse and patchy hair coat, multiple to generalized distribution of raised plaques with overlying scales and ulceration, a generalized erythema, and erosion of the oral mucosa and mucocutaneous junction. Complete blood count (CBC), coagulation panel, and urinalysis collected by void were relatively unremarkable with no significant abnormalities. Serum chemistry panel (CHEM) demonstrated an elevated cholesterol (297 mg/dl), ALT (325 U/L), and ALKP (420 U/L). Three-view thoracic radiographs identified mild left atrial enlargement with no evidence of cardiogenic pulmonary edema and no evidence of pulmonary neoplasia identified. Sonographic assessment of the abdomen revealed liver enlargement consistent with steroid hepatopathy, renal dystrophic mineral and possible calculi, and bladder calculi and possible cystitis. Fine-needle aspiration of mandibular, superficial cervical, popliteal, and inguinal lymph nodes indicated reactive lymphoid hyperplasia. Due to the patient’s prior and persistent hepatotoxicity secondary to CCNU, they were excluded from participation at the time of initial evaluation. At the client’s perseverance, lack of response to prior therapy, and progressive disease, the patient was referred from medical oncology to radiation oncology for TSPT. Delivery of radiation therapy commenced approximately 30 days (30 days) after initial presentation with no additional staging prior to start.

### 3D mold generation

**Fig. 1:**
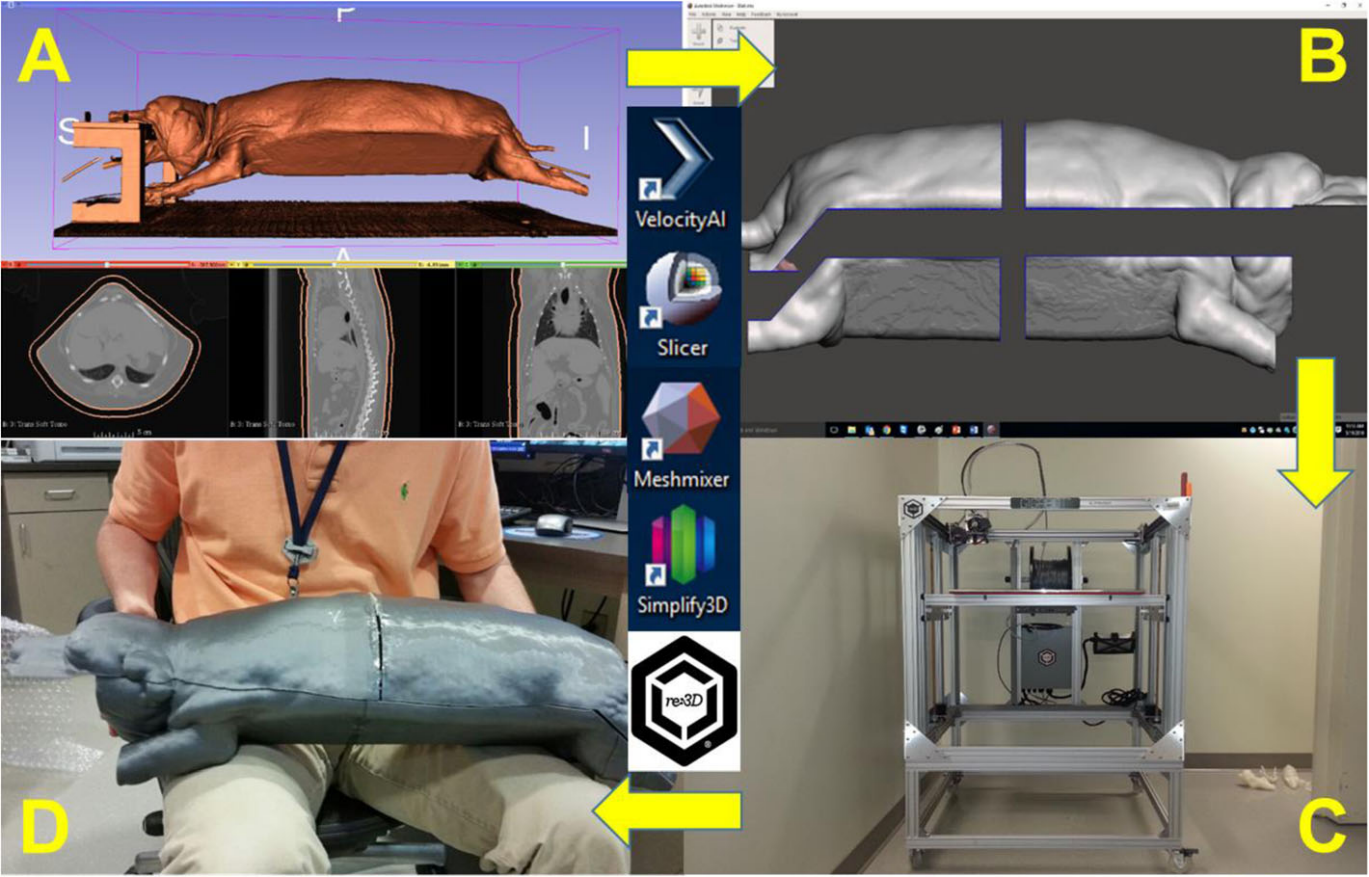
3D mold generation and workflow. **a** The patient’s surface contour and 10 mm expansion into air generated off a whole body CT image set. **b** 3D mesh transformation and segmentation into four separate interconnected shell components. **c** Stereolithographic file exported to an example 3D printer for fabrication. **d** Patient’s printed shell assembled and ready for clinical use

An indexible nylon Vac-Lok™ cushion (CIVCO Medical Solutions, Coralville, Iowa) immobilization system and an in-house, canine-specific bite block fixation device were used to rigidly immobilize the head and neck, body, and limbs. For 3D mold generation, a computed tomography (CT) image set of the whole body was acquired. The entire patient was scanned in a large bore (80 cm) CT scanner (Siemens Somatom Definition AS). The image set was transferred to a VelocityAI (Varian Medical Systems Inc., Palo Alto, CA.) workstation for contouring. To generate the initial 3D mold scaffold, the patient’s surface was contoured and expanded 10 mm into air (Fig. 1a). To prepare the 3D mold scaffold for final printing, the 3D mold contour was then transformed into a 3D mesh using 3DSlicer [22], segmented into four separate interconnected shell components using Meshmixer (Autodesk, San Rafael, CA.) converted to a stereolithographic file for 3D printing using Simplify3D (Cincinnati, OH.), and sent to re:3D (Austin, TX.) for manufacturing/printing (Fig. 1b-d). The mold was fabricated using polylactide (PLA) filament with a mass density of approximately 1.09 g/cm^− 3^ and an infill percentage of 100.

### Helical tomotherapy planning

**Fig. 2:**
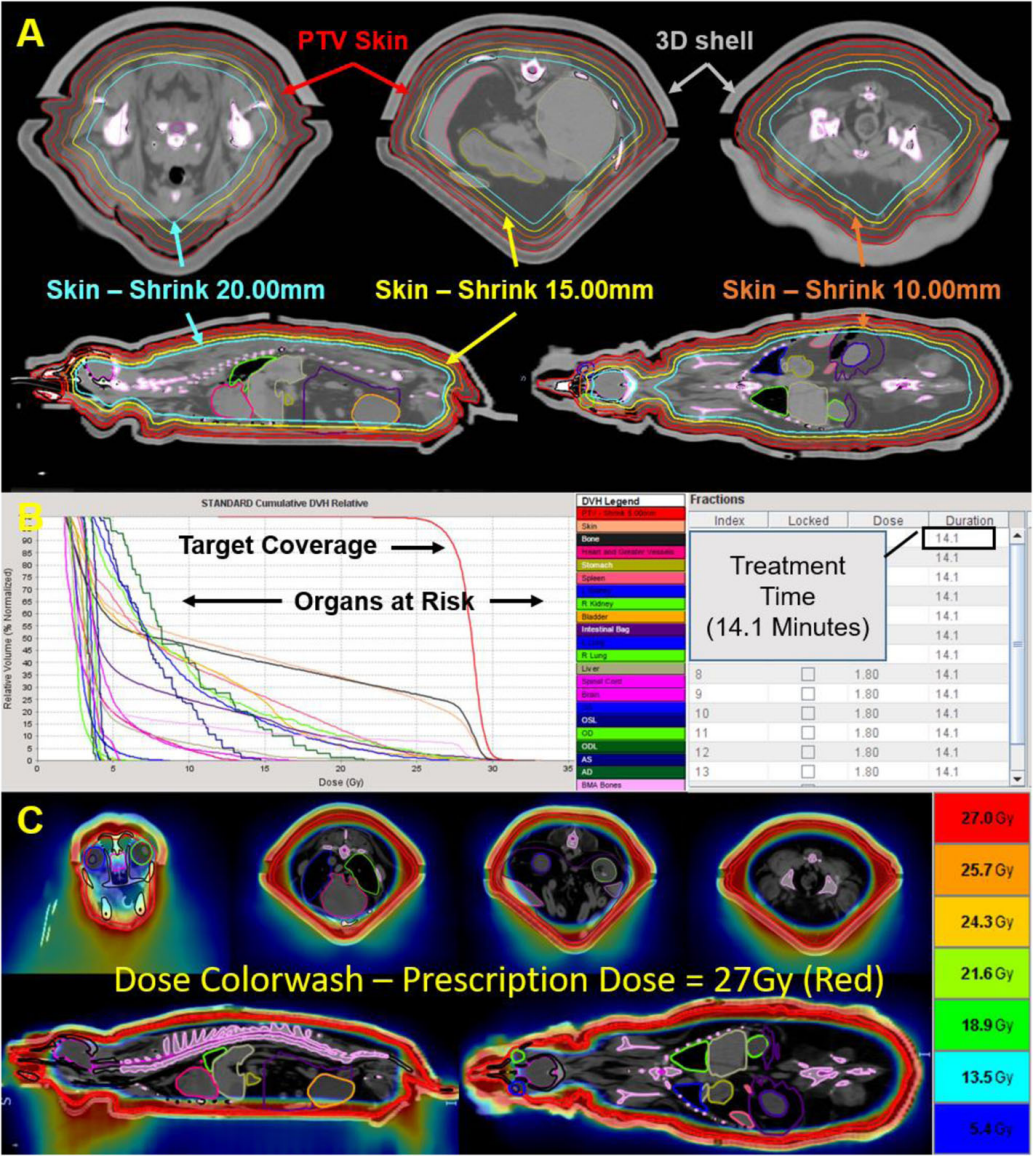
Helical tomotherapy planning workflow and evaluation. **a** Patient contained within 3D printed mold. Three planning contours (10 mm, 15 mm, and 20 mm expansions inward from the subcutaneous side of the PTV) were used to control dose to the core of the patient. **b** The dose volume histogram (DVH) of the target, individual organs at risk (OARs), and actual beam-on treatment time. **c** Colorwash of dose distribution within the patient

For helical tomotherapy treatment planning (v. 4.0.4. Tomotherapy, Inc., Madison, WI) a CT image set of the patient’s whole body encased within the 3D printed mold was acquired. The image set was transferred to a VelocityAI workstation for target and normal tissue contouring. Lungs, heart, liver, kidneys, spleen, intestines, stomach, bladder, brain, spinal cord, eyes, estimated bone marrow cavities (cervical vertebrae/caudal skull, thoracic vertebrae/ribs/sternum, abdominal vertebrae/pelvis, brachium and scapula, and femur) thyroids, and lenses (among other volumes) were contoured as organs at risk (OAR). The clinical target volume (CTV) included the entire body surface and extended 3 mm subcutaneously. To account for setup variability and the impact of respiratory motion, the CTV was expanded 2 mm isotropically to form the planning target volume (PTV). Three constraint contours (10 mm, 15 mm, and 20 mm expansions inward from the subcutaneous side of the PTV) not representing OARs or target structures but used strictly as planning tools for dose optimization were generated for dose constraints to the core of the body (Fig. 2a).

Initially, 27 Gy delivered in 15 fractions, 4 times per week were prescribed to 92% of the PTV. Normal tissue dose constraints where based on previously reported clinical tolerances to various OARs. The field width, pitch, and modulation factor used for treatment planning optimization were 5.0 cm, 0.430, and 3.5 respectively. Dose volume histograms and isodose lines were evaluated for the target and individual OARs (Fig. 2b-c). Normal tissue toxicity from treatment was evaluated and scored according to the Veterinary Cooperative Oncology Group – Common Terminology Criteria for Adverse Events v1.1 [23]. Due to hematological toxicity after fraction 8, a treatment interruption was instituted to allow for recovery and the prescription and frequency was modified. The remaining 7 fractions had the dose per fraction reduced from 1.8 Gy to 1.4 Gy (plan not shown) and to compensate for the loss of biological effect from fraction size and treatment break, the number of remaining fractions was increased from 7 to 9.

### Image guidance

**Fig. 3:**
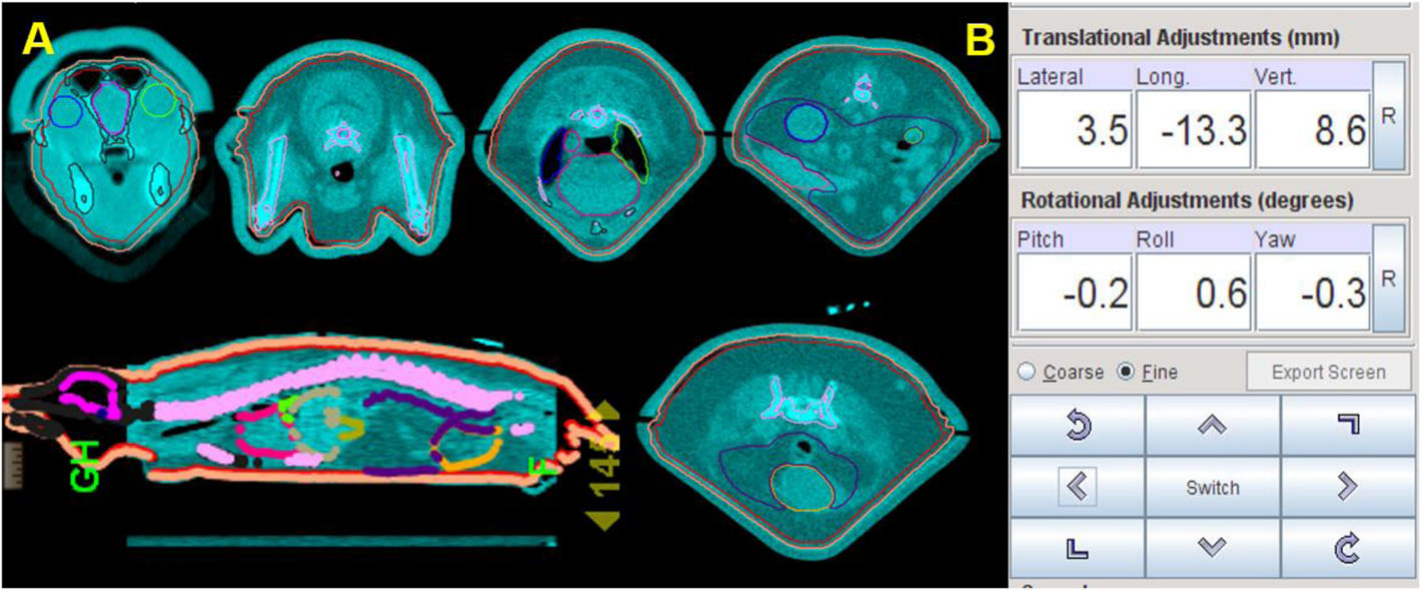
Patient setup verification using MVCT image guidance. **a** MVCT image fusion with treatment plan contours prior to treatment delivery. **b** Patient registration shifts to correct for misalignment between planning CT and MVCT prior to treatment delivery

Patient positioning and setup was verified by onboard volumetric megavoltage CT (MVCT) system integrated in the helical tomotherapy machine. Daily MVCT scans (approximately 3 cGy to the body regions for each daily scan) were performed cranially from the level of the eyes caudally to the rear limbs. Image fusions were evaluated by the radiation oncologist and any appropriate translational shifts were applied to the patient’s setup prior to treatment delivery (Fig. 3).

### Anesthesia

The patient was pre-medicated with butorphanol (0.2 mg/kg IV) and induced slowly to effect with propofol (2–4 mg/kg IV) for each treatment. The patient was intubated and placed on Sevoflurane (2.5–3%) in oxygen. Intravenous fluids (Lactated Ringers Solution) were provided through the cephalic catheter throughout anesthesia (6 ml/kg/hr). Manual ventilation was provided until the patient had been fully positioned within the body mold then the patient was switched to mechanical ventilation at 17 breaths/min, 10 ml/kg and inspiratory pressure of 19 cm H20 (Hallowell Ventilator). Monitoring included a pulse oximeter probe on the tongue, oscillometric blood pressure cuff on the forelimb, ECG patches on the ventral paw pads and side stream EtCO2 (Vetrends MAX multi-parameter monitor). The body mold did not extend past the proximal forelimb or distal to the hock to allow placement of the IV catheters, ECG patches and oscillometric cuff. Weekly NOVAs (Waltham, MA) were run prior to the start of each week of radiation therapy to ensure patient candidacy for anesthesia. Due to the localization of the disease to the skin only (as determined by prior staging) and the obesity of the patient, an ASA physical status score of 3 was initially assigned.

### Dose measurement

Patient-specific delivery quality assurance was performed in a solid-water phantom native to the system using Radiochromic EBT3 film (Ashland, Covington, KY.) and ionization chamber measurements to verify the planned fraction delivered dose (not shown). Relative planar dose profiles and absolute point dose measurements were compared to calculated planar isodose profiles and point doses. Tolerance for the plan to be deemed acceptable was +/− 3% for measured point doses and gamma value </= 1 for 90% of all points lying within the 30% isodose line using search criteria of 3% and 3 mm. Six nanoDot™ (Landauer, Glenwood, IL) dosimeters were placed along the dorsal and ventral surfaces of patient midline as secondary in vivo verification of dose received to the surface of the skin. Several types of dosimeters could have been considered for in vivo measurements, but the size, ease of appositional placement, processing, and independent accuracy of nanoDot™ thermoluminescent dosimeters made them ideal. Additionally, the uncertainty in dose inherent to the dosimeters is well under the variation that classical TSEBT patients experience day to day and patient to patient variation [12, 24].

### Response and toxicity

**Fig. 4:**
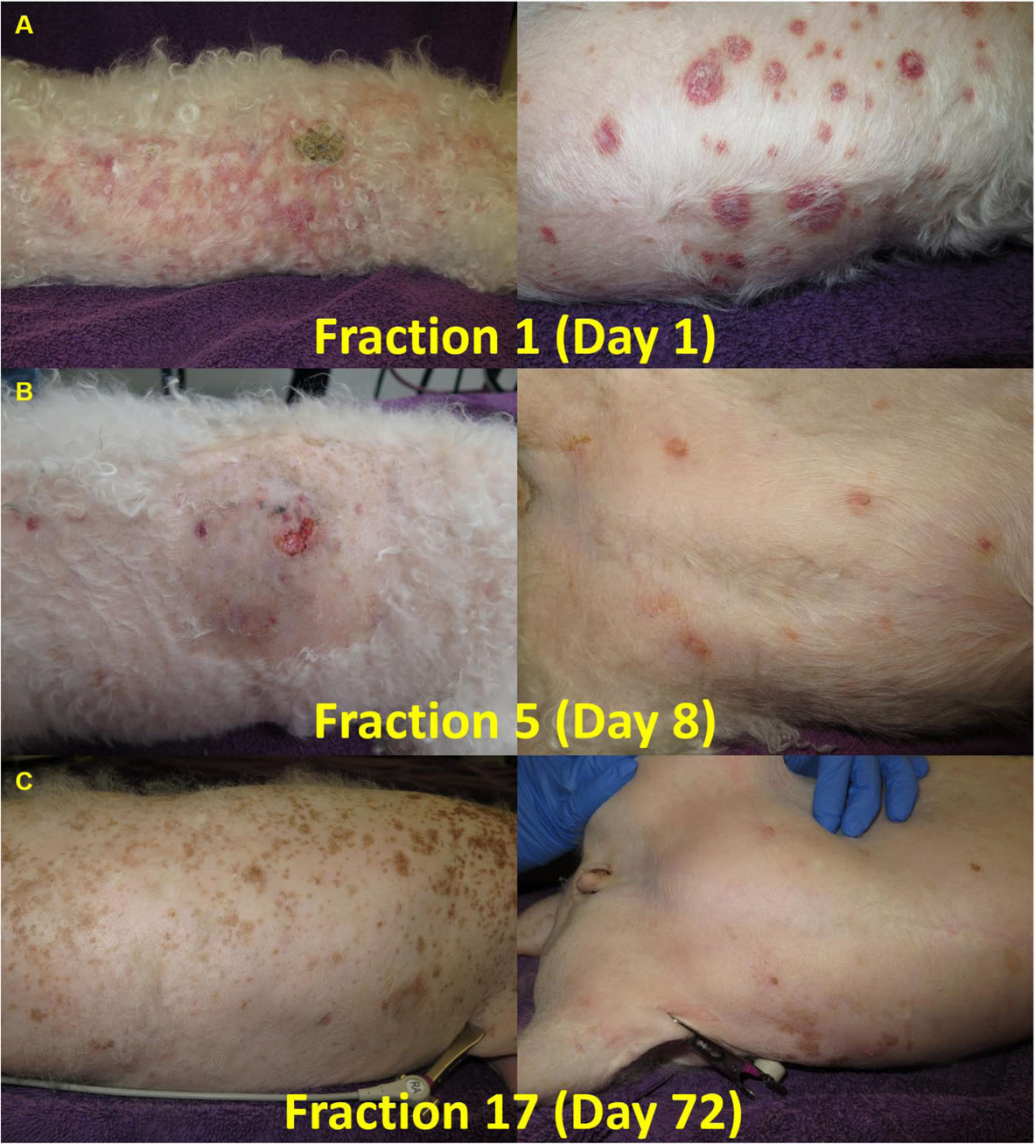
Treatment response over the course of therapy. **a** Example of patient’s disease burden prior to the first fraction of total skin radiation. **b** There was noticeable improvement by the completion of the fourth treatment. **c** The tumor regressed progressively over the entire body without further noduloplaque progression by completion of the protocol

Twenty-seven gray were delivered to the patient from July 13th, 2015 to September 23rd, 2015. A partial response was noticeable after four fractions and the tumor completely regressed over the entire treated area by the end of therapy (Fig. 4a-c). No follow-up histology for pathological response was performed at the request of the client. Grade 1 lethargy, fatigue, weight loss, and oral mucositis and grade 2 alopecia, nail/claw changes, pruritus, scaling, anorexia, and diarrhea were noted during and within a couple weeks of treatment. Additionally, grade 3 thrombocytopenia developed after fraction eight requiring a treatment interruption of 6 weeks and prescription modification prior to treatment continuation and completion (Tables 1, 2, 3, 4 and 5). No clinically relevant abnormal liver or renal functions and, although not specifically assayed, there was no clinical sign of thyroid or pituitary axis dysfunction detected during or after treatment and the follow-up period prior to the event leading up to the time of euthanasia. Supportive measures were provided and all toxicities except alopecia and thrombocytopenia which fluctuated between grade 1 and grade 2 fully resolved without further incident. Transient pyoderma was noted on follow-up examines after treatment. From the beginning of TSPT treatment until the time the patient was euthanized unrelated to CEL (complications associated with acute pancreatitis) on November 13th, 2015 (approximately 123 days), only one new lesion on the head was detected and confirmed by histopathology within the treated field.

**Table 1:** Treatment related toxicity as characterized by the Veterinary Cooperative Oncology Group - common terminology criteria for adverse events following biologic antineoplastic therapy in dogs and cats v1.1

Category	Adverse Event	Grade	AE Attribution
Bone Marrow	Thrombocytopenia	Grade 3	Definite
Constitution	Lethargy	Grade 1	Definite
	Fatigue	Grade 1	Probable
	Weight Loss	Grade 1	Possible
Dermatological	Alopecia	Grade 2	Definite
	Nail/Claw changes	Grade 2	Definite
	Pruritus	Grade 2	Definite
	Scaling	Grade 2	Definite
Gastrointestinal	Oral Mucositis	Grade 1	Definite
	Anorexia	Grade 2	Possible
	Diarrhea	Grade 2	Possible

**Table 2:** CBC values 30 days prior to the start of treatment, during treatment, treatment holiday nadirs, last day of treatment, and 30 days post completion of TSPT

Hematology	Day −30 Initial	Day 1 (FX 2)	Day 10 (FX 8)	Tx Holiday (Days 11–54) (Nadirs)	Day 53 (FX 9)	Day 72 (FX 17)	^a^Day 102 + 30 Days PostRT
AbsoNeut 3000–11,500 (WBC k/ul) 6.0–17.0	7896 (8.4)	14,852 (15.8)	11,305 (11.9)	4416 (4.6) (Day 22)	8281 (9.1)	13,485 (14.5)	8445 (10.4)
RBC (M/ul) 5.50–8.50	5.38	4.73	4.86	4.53 (Day 22)	6.00	5.75	6.31
Platelet (/ul) 200,000–500,000	362,000	418,000	193,000	36,000 (Day 35)	324,000	180,000	Adq to decreased

^a^Day 102 bloodwork was submitted for analysis to Idexx Laboratories (Totowa, NJ) by the rDVM. Platelets were evaluated by manual review and interpreted to be between 100,000 to adequate. Large platelets were noted

**Table 3:** Serum chemistry values 30 days prior to the start of treatment, during the treatment holiday, and last day of treatment

Chemistry	Day −30 Initial	Tx Holiday (Day 35)	Day 72 (FX 17)
PHOS (mg/dl) 2.9–6.2	5	5.9	6.6
GLU (mg/dl) 60–135	103	95	81
LAC (mg/dl) 9.9–46.8	17.9	22.4	30.5
CHOL (mg/dl) 120–247	297	260	272
BUN (mg/dl) 5–29	13	26	17
CREA (mg/dl) 0.3–2.0	0.52	0.55	0.59
Mg (mg/dl) 1.7–2.1	1.7	1.6	2.2
Ca (mg/dl) 9.3–11.8	10.8	10.6	10.4
TP (g/dl) 5.7–7.8	6.1	5.9	5.7
ALB (g/dl) 2.4–3.6	3.4	3.3	2.9
GLOB (g/dl) 1.7–3.8	2.8	2.6	2.8
ALT (U/L) 10–130	325	61	180
ALKP (U/L) 24–147	420	235	729
GGT (U/L) 0–25	12	< 10	< 10
TBIL (mg/dl) 0.0–0.8	< 0.1	< 0.1	< 0.1
Na + (mmol/L) 139–147	148	144	146
K+ (mmol/L) 3.3–4.6	3.5	4.4	3.8
Cl- (mmol/L) 107–116	112	108	109
ECO2 (mmol/L) 21–28	22	23	26
AGPK (mmol/L) 10–18	17.2	17.4	14.9

**Table 4:** Urinalysis values 30 days prior to the start of treatment and during the treatment holiday

Urinalysis	Day −30 Initial	TX Holiday (Day 26)
Color	lt yel/ clear	lt/yellow clear
SPGR 1.015–1.045 GMS/100 ml	1.020	1.025
PH 6.000–7.000	6.000	7.000
PRO NEG - Trace mg/dl	neg	N
GLUC mg/dl	neg	N
KETO	neg	N
BILI	neg	N
BLOOD NEG - NEG	neg	N
UROB 0.100–1.000 mg/dl	0.200	0.200

**Table 5:** Coagulation values 30 days prior to the start of treatment and during the treatment holiday

Coagulation	Day −30 Initial	TX Holiday (Day 18)
PT 6.0–7.5 s	6.9	6.7
PTT 7.1–10.0 s	9.7	9.3
PTFIB 64–202 mg/dl	490	776
FIBC 116–364 mg/dl	609	1002
ATIII 114 - % NHP	99.5	102
D-DI 116.2–371.5 ng/ml	305	280

### Organs at risk

The min, mean, and max doses of TSPT to various OARs are presented in Table 6.

**Table 6:** Planned doses to be delivered to organs at risk during total skin photon beam radiation

Organ at Risk	Min Dose (Gy)	Mean Dose (Gy)	Max Dose (Gy)
L Middle Ear	3.59	4.02	4.73
R Middle Ear	3.12	3.37	3.84
Bladder	1.98	9.33	30.23
Bone Marrow Avoidance (Abdomen/Pelvis)	2.01	3.13	19.85
Bone Marrow Avoidance (Cervical)	1.78	5.01	28.77
Bone Marrow Avoidance (Femurs)	1.76	7.49	29.56
Bone Marrow Avoidance (Forelimbs/Scapulae)	2.18	12.31	29.21
Bone Marrow Avoidance (Thoracic Vertebrae/Ribs)	1.76	5.02	28.77
Bone Marrow Avoidance (Union- All)	1.76	5.94	29.21
Whole Brain	2.85	4.73	17.4
Heart	1.94	4.02	13.28
Intestinal Bag (Intestines)	1.88	7.5	30.77
L Kidney	2.91	3.54	4.22
R Kidney	2.65	3.71	4.31
L Lung	2.08	3.05	9.19
R Lung	1.97	2.69	7.57
L Thyroid	9.04	15.04	21.6
R Thyroid	8.96	15.22	23.73
Liver	2.03	4.37	27.58
L Eye	3.69	9.51	26.88
L Eye, Lens	4.14	7.85	15.15
R Eye	3.41	9.97	28.88
R Eye, Lens	4.55	9.97	19.42
Pituitary Gland	3.59	4.26	5.02
Spinal Cord	1.77	2.91	5.45
Spleen	2.61	10.03	28.73
Stomach	2.05	3.16	5.08
Pancreas	0.71	0.74	0.8

### Independent dose verification to the surface of the skin

**Fig. 5:**
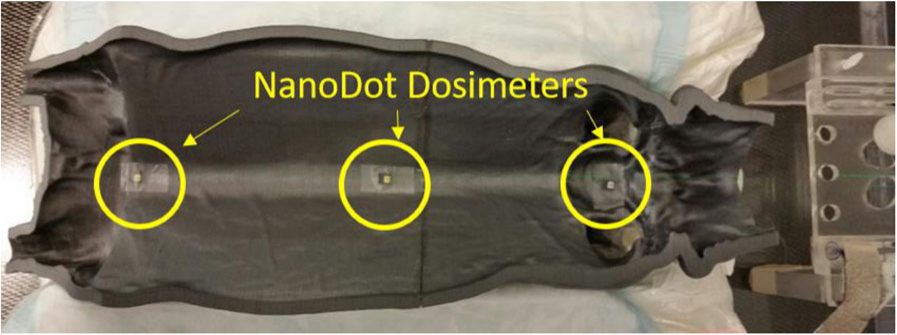
Ventral nanoDot™ placement for in vivo dosimetry and secondary verification prior to the first administered fraction of total skin radiation

The surface dose to the skin as verified by the placement of six nanoDot™ dosimeters along the dorsal and ventral surface of the patient are listed in Table 7. Ventral placement of the dosimeters prior to the first treatment fraction is demonstrated in Fig. 5.

**Table 7:** Secondary dose verification to the surface of the skin at the time of the first delivery, nanoDot™ dosimeters were positioned in areas where classical TSEBT uncertainties would present or at mold intersections where a gap may exist

Site	NanoDot Serial Number	Surface Dose (cGy)/Fraction	%D Prescription Dose
Dorsal Head (Vertex)	DN086200581	180.684	0.38
Dorsal Thorax (T6-T7)	DN08620795P	180.991	0.55
Dorsal Abdomen (Sacrum)	DB08305757G	180.584	0.32
Ventral Neck (Larynx)	DN09050308Y	176.349	−2.03
Ventral Thorax (Xiphoid)	DN08749836D	179.259	−0.41
Ventral Abdomen (Inguinal)	DN08749729A	180.181	0.10

Prescription Dose: 180.00 cGy. Uncertainty of NanoDot measurement: +/− 1.7%

## Discussion and conclusion

The design for this technique of TSPT for treating canine companion animal consisted of two main goals: create a time efficient and clinically implement technique and use 3D printing technology to address the issues plaguing TSEBT dose homogeneity and reproducibility. The TSEBT technique in canine companion animals has been deemed mechanistically feasible, however prior attempts to clinically implement successfully required protracted time commitments from the patient and staff [13, 16]. As reported, several hours were necessary to deliver a dose of 2.5 Gy to each of the 12 treatment sub fields. While times will depend on total treatment volume (patient size), the setup and treatment time for TSPT utilizing 3D printing technology reduced that time requirement down to under 40 min including real time monitoring of anesthesia during setup and treatment. Since the 3D printed mold is generated from the patient’s surface contour, patient reintegration into the mold at the time of treatment is time efficient as the patient fits consistently in place day after day. Of course careful attention to patient nutrition is paramount as body volume needs to be well maintained in order to assure good mold/skin apposition. Additionally, setup reproducibility is improved because the mold is rigid, indexible with immobilization equipment, and contains the patient within, resulting in the downstream effect that if the mold is in place (verifiable through volumetric imaging) and the patient is repositioned correctly within (also verifiable through volumetric imaging), then for delivery, the patient is in the correct place.

TSEBT, as expected, creates extreme dose variation on and around extremities and where surface contours change abruptly or overlap. Just like in humans, canine companion animals experience hot and cold spots found near the axillary and inguinal areas, perineum, tail, top of the head, plantar and palmar surfaces of the pez and manus, muzzle, midline sternum, between the eyes, and midline inguinal area. Uneven dose distributions necessitate shielding or patch fields to compensate all adding additional setup and treatment time. Dose heterogeneities reported in canine companion animals range from − 58.4 to + 73.9% from the intended prescription dose introducing the possibility for significant tumor underdosing or normal tissue overdosing [16].

TSPT takes advantage of photon interactions with matter and are not as sensitive as electrons to surface contour perturbations. Additionally, heterogeneity is reduced as helical tomotherapy can modulate beam intensity and requires no field matching when the treatment length is under 160 cm which encompasses a majority of the canine patients treated. However, unlike electrons which have a limited range of penetration determined by nominal energy chosen, photons have a probability of interacting which means treating directly en face results in clinically lethal doses to the core of the body.

TSPT technique addresses this issue by restricting the use of only tangential treatment beams thus minimizing the dose which reaches organs at risk. Restricting dose delivery to exclusively tangential beamlets can provide adequate superficial doses but is sensitive to positional changes (patient setup and motion) and treatment planning system optimization algorithms (accurate superficial doses approximations). Authors have found significant impact on surface dose using helical tomotherapy where PTV extended up to or close to the surface of the skin [25–27]. In one study, film measurements in the superficial region showed that the helical tomotherapy treatment planning system overestimated the dose by 14% at a 1 mm depth, improving to 3% at depths greater than 5 mm [28]. In order to reduce the impact of skin to air interface changes in the dose calculation during optimization, the mold was constructed to be 10 mm thick of near tissue equivalent material. Utilizing the 3D printed mold of the patient also reduces setup uncertainty and reduces motion. Veterinary radiation oncology patients treated at Texas A&M are under general anesthesia with mechanical ventilation. Respiratory motion is mechanically limited in patients encased within the mold. In order to reduce the impact of a force-reduced tidal volume, the patient had an increase in respiratory rate and decrease in tidal volume to maintain adequate minute volume. Despite these adjustments to ventilation the patient maintained an EtCO2 and inspiratory pressure slightly above normal at 50 mmHg and 19 cmH20 respectively. A novel ventilation technique such as high frequency oscillatory or jet ventilation may have provided better ventilation parameters as well as decreasing respiratory motion impact during imaging and treatment.

In humans, common toxicities happening at the time or shortly thereafter from TSEBT include pruritus, dry desquamation, erythema, alopecia, bullae of the feet, edema of the hands and feet, hypohidrosis, hyperpigmentation of skin, phlyctenules, and loss of fingernails and toenails. Long-term complications are typically mild but may include permanent nail dystrophy, xerosis cutis, alopecia, and digital dysesthesia [29]. While dose delivered to organs at risk with the TSPT technique where higher than classical dose amounts given in human patients, the amounts were well within clinical tolerances for each structure individually. Short and long-term toxicity profiles experienced in our patient were well within these expected limits with the exception of the grade 3 thrombocytopenia. Hematological toxicity is uncommon with TSEBT because of the limited depth of penetration of the electrons and minimal photon contamination in the beam. Unexpected hematological toxicity has been reported in humans using a similar technique using a neoprene wetsuit [30, 31]. A Grade 3 hematological toxicity experienced in TSPT by our patient was unexpected but the grade is more than likely an intricate interplay between hematopoietic reserve (how heavily pretreated they were), technique refinement (more modular mold, better ventilation technique, etc.), and time-dose fractionation chosen (more appropriate dose per fraction and number of fractions per week). When the patient presented to Texas A&M, they had received several systemic forms of cytotoxic therapy all potentially impacting functional hematopoietic reserves. It’s quite possible their rebound capability was partially blunted by insult from preexisting therapies. From a technique perspective, virtual studies were performed and all foreseeable issues (within clinical constraints) where addressed prior to clinical implementation, however, there were still several unknowns which where dynamically adjusted for during treatment. Experienced gained during each fraction of treatment were applied to aid in the optimization of future fractions delivered. From a target dose perspective, our prescription fell within clinically used doses in human TSEBT patients, however, the dose to our organs at risk, albeit, still clinically acceptable by individual organ standards in veterinary and human medicine, were higher than what is classically considered acceptable for TSEBT in humans. In the future, the planning constraints for our organs at risk should be stricter and our prescription should be less aggressive (fraction size less than 1.8 Gy starting with no more than three fractions per week) to reduce the possibility of higher grade hematological damage.

This TSPT technique utilizing a 3D printed shell provides precise time-efficient dosage delivery, improved dose characteristics to the target and OARs. Additionally, although care needs to be taken when interpreting the results of a single patient’s response, impressive results were seen especially given the refractory nature of this patient’s disease. For dedicated clients, the proposed technique could be considered an acceptable alternative to TSEBT (which is not practically implemented in veterinary medicine) that is actually clinically implementable (outside of a palliative setting) within the right academic setting (delivery system, physics support, and fabrication requirements), however further testing and refinement is needed to reduce hematological complications and confirm preliminary findings.

## Data Availability

All data generated or analyzed during this study are included in this published article [and its supplementary information files].

## Abbreviations

ASA: American Society of Anesthesiologist
CEL: Cutaneous epitheliotropic lymphoma
CHOP: Cyclophosphamide, hydroxydaunorubicin, vincristine, prednisone
CT: Computed tomography
CTV: Clinical target volume
Gy: Gray, J/kg
IMRT: Intensity-modulated radiation therapy
MOPP: Mechlorethamine, vincristine, procarbazine, prednisone
MV: Megavoltage
MVCT: Megavoltage computed tomography
OAR: Organs at risk
PLA: Polylactide filament
PTV: Planning target volume
TSEBT: Total skin electron beam radiation therapy
TSPT: Total skin photon beam radiation therapy
